# Cross-sectional survey of knowledge and attitudes of healthcare workers and community members toward the Ebola virus disease and antimicrobial resistance pathogens outbreaks in Nigeria

**DOI:** 10.11604/pamj.2021.40.116.20561

**Published:** 2021-10-22

**Authors:** Ibrahim Yusuf, Auwalu Halliru Arzai, Muhammad Yusha´u, Lawal Garba, Musa Haruna, Muhammad Ibrahim Getso

**Affiliations:** 1 Department of Microbiology, Faculty of Life Sciences, Bayero University, Kano, PMB 3011, Kano, Nigeria,; 2 Department of Microbiology, Gombe State University, Gombe, Nigeria,; 3 Department of Biology, Kano University of Science and Technology, Wudil, Kano, Nigeria,; 4 Department of Medical Microbiology and Parasitology, Bayero University, Kano, PMB 3011, Kano, Nigeria

**Keywords:** Ebola, antimicrobial resistance, pathogens, health care workers, community members, awareness, knowledge, attitude

## Abstract

**Introduction:**

the 2014 Ebola virus disease (EVD) outbreak in Nigeria has further raised the awareness of health-care workers (HCWs) and community members (MCs) on the threat posed by infectious diseases and the need for improvement on infection control practices. However, awareness of dangers of increasing incidences of antimicrobial resistance (AMR) in hospitals and communities remained low.

**Methods:**

a cross-sectional survey of awareness of 195 HCWs and 265 MCs toward EVD and AMR was conducted through a structured questionnaire.

**Results:**

majority of HCWs (95.4%) and MCs (82.8%) still have knowledge of EVD´s danger and give reasons like its unique way of killing and unavailability of drugs for their awareness. Only 17.2% of MCs are aware of AMR as a problem, and only 3.4% of MCs and 10.3% of HCWs agreed that AMR is more dangerous than EVD. On the contrary, 76.4% of doctors, 95.1% nurses, 67.9% laboratory scientists, 66.7% pharmacists, 77.4% students and 100% of civil servants, drivers and religious leaders believed that EVD is more horrific and spread faster. They both attributed the rapid awareness of EVD in Nigeria, despite being new at the time of the outbreak, to the seriousness with which stakeholders and the media fought EVD, the gesture AMR is yet to receive. Though both HCWs and MCs agreed that prevention, not treatment is the best option to tackle Ebola like-diseases, but surprisingly, about 37% and 65% of HCWs and MCs respectively, still believe that traditional medicines can be used to treat Ebola related illnesses.

**Conclusion:**

AMR awareness remains low among MCs and some HCWs when compared with EVD. It is recommended that efforts put in place during EVD outbreak by all stakeholders and the media need to be doubled to increase the knowledge of both HCWs and MCs toward AMR.

## Introduction

Ebola viral disease (EVD) and infectious diseases due to antibiotic-resistant pathogens (ARPs) are becoming an epidemic in many African countries [1-3]. The treatments of the duo are either costly or unavailable, therefore can lead to high morbidity and mortality, especially in developing countries where adequate infection tracking and control systems are inadequate [4,5]. A single individual infected with EVD or ARP in a health-care facility or community can put others in the hospital or the community at higher risk of infections [6,7]. Even though there is scanty information on the level of antimicrobial resistance (AMR) among different clinical isolates in Nigeria and other African nations due to the lack of effective systems of surveillance and reporting, a recent estimate [3] indicated a significant increase in resistance and spread of bacteria such as *Escherichia coli, Staphylococcus aureus*, and *Klebsiella pneumonia* in many hospitals and communities in the region [8]. A high level of resistance to cephalosporin and fluoroquinolones by *E. coli* was common and more frightening is the fact that a substantial number of *Staphylococcus aureus* infections are caused by methicillin- resistant (MRSA)- strains that are virtually difficult to treat [8].

The most common ARPs in Nigeria include MRSA, Vancomycin-resistant *Staphylococcus aureus/Enterococci*, Extended-spectrum beta-lactamase (ESBL), and carbapenemase-producing Gram-negative bacteria [9,10]. Studies have indicated that approximately 25 percent of all clinical death in Nigeria is caused by drug resistance [11] while, the total confirmed EVD deaths in the recent and only outbreak in Nigeria is 8 out of the total 11, 325 (0.07%) confirmed deaths in Sub Saharan Africa [12]. The indisputable fact that the number of deaths due to AMR are far ahead of the number of deaths due to EVD in Nigeria remained unnoticed among many health-care workers (HCWs) and members of the community (MC) and one health approach designed to effectively address AMR issue is at least not yielding expected result at present. Even though the etiological agents of EVD and AMR differ, they share a similar mode of transmission, which is through unhygienic practices and direct contact. Based on the disparity in mortality figures and similarity in the means of transmission, it is expected that AMR should receive more attention from HCWs and MCs than EVD, but the way media portrayed EVD and other infectious diseases such as COVID-19 in Nigeria and other African nations has placed it´s danger ahead of AMR in the heart of many HCWs and MCs, while major health threats posed by AMR and ARPs remain insufficiently newsworthy.

A significant number of EVD and ARP infections are spread by HCWs, especially in developing countries where infection control and occupational safety is a neglected issue [13-15]. While active knowledge of the danger of EVD among HCWs and MCs has played a significant role in curbing its spread in Nigeria during the 2014 outbreak [16], the level knowledge of AMR and ARPs among HCWs and MCs and how it plays role in curbing ARP´s spread is unknown. EVD literature is replete with published data that evaluated the knowledge, attitude, and practice of HCWs on EVD [4,17-21]. However, no investigation had compared the awareness level of HCWs and MCs toward EVD and AMR in relation to controlling the spread of the duo in Nigeria. Owing to the alarmingly increasing spread of AMR in Nigeria [10,17,18], this study evaluated the awareness level of HCWs and MCs toward EVD and AMR. The study was planned to evaluate the HCWs´ knowledge and attitude toward EVD and AMR, their dangers, how they are transmitted, their prevention and control methods, and to compare it with that of MCs. We postulated that there would be a significant difference in awareness levels of HCWs and MCs toward EVD and AMR.

## Methods

**Study design:** the design of this study was cross-sectional and exclusively non-experimental. The data were obtained through a structured questionnaire-based interview from HCWs and MCs in 10 health-care facilities situated in Kano state, North West, Nigeria.

**Ethical approval:** before the start of this study, approval from the concerned authorities of the Kano State Hospital Management Board was obtained (MOH/Off/797/T.I/286).

**Participants:** for this study, HCWs are considered professional individuals who work in the hospital and have direct or indirect contact with patients. Therefore, doctors, nurses, pharmacists, medical laboratory scientists, medical laboratory technicians (MLTs), and community health workers (CHEW) were HCWs chosen to complete the questionnaire. Similarly, MCs are considered any individual who is not HCW and works outside the hospital. MCs involved in this study are categorized as students, civil servants, drivers, religious leaders (imam and pastors), traditional healers, and rural dwellers (villagers/farmers). HCWs were recruited randomly from 10 healthcare facilities across the state while MCs were sought from the communities where the health-care facilities were located.

**Questionnaire:** to evaluate the knowledge and attitude of HCWs and MCs on EVD and AMR/ARPs, a well-structured, pretest questionnaire was used to collect the information needed. A pilot study was conducted on 15 HCWs and 25 MCs that were excluded from the study. After the pilot study, the content of the questionnaire was restructured, where the clarity of some questions and responses were improved and some were deleted. The questions were devised loosely based on WHO infection prevention and control (IPC) guidelines for EVD and AMR, with further appropriate questions relevant to the level of understanding of the MCs. The final questionnaire comprised 4 main sections. The sections comprised questions on general knowledge of EVD and AMR, comparison of EVD and AMR dangers, the transmission of EVD and ARPs, prevention of EVD and ARP spread. Some questions are fixed-choice items in the form of a Likert scale, while others are in the form of multiple-choice questions. The demographic information collected from all participants is age, gender, and occupation. However, profession, occupation, and years of experience were also included. Other parts of the questionnaire retrieved information from both HCWs and MCs on previous knowledge of EVD and AMR, their means of transmission, sources of information on EVD and AMR, comparison of dangers of EVD and AMR, attitudes toward prevention and control of EVD and AMR, and general treatment of EVD and ARP's infections. A total of 600 structured questionnaires were then distributed to 300 HCWs and 300 MCs by research assistants after obtaining their consent. The purpose of the study was explained to all the respondents in the English language, but for MCs that do not understand English, the content was communicated to them in their local dialect by an experienced research assistant, and the questionnaire was filled according to their chosen options. Assurance of the confidentiality of their personal data and other information shared was also provided.

**Data analysis:** from the information retrieved, a comparative analysis was conducted between HCWs and MCs, which allowed us to identify trends both between and within groups. The results are presented in frequency distribution tables. Statistical analysis was performed using the SPSS statistical package (SPSS Inc., Chicago, IL, USA). Chi-square test was used to compare differences between groups. Statistical significance was set at p < 0.05.

## Results

**Study population:** of the total six hundred questionnaires distributed to both the HCWs and MCs, a total of four hundred and sixty (460) questionnaires were completed. One hundred and ninety-five were completed by HCWs, while two hundred and sixty-five were completed by MCs. The response rate of HCWs was 65% while that of MCs was 88.3%. One hundred and five HCWs and thirty-five MCs did not return their questionnaires despite repeated visits. Of the completed questionnaires, thirty (30) including 2 doctors, 4 nurses, 9 MLS, 7 MLT, and 8 pharmacists´ responses were excluded from the analysis because they did not fill the demographic section. On the MC's side, 16 respondents, which comprised 3 students, 5 drivers, 1 religious leader, and 7 villagers did not respond to the questions after their consent was initially sought and eighteen respondents (9 drivers, 6 villagers, 2 civil servants, and 1 student) only filled the demographic section. Besides, 41 MCs respondents (4 butchers, 6 hawkers, 6 bricklayers, 4 laborers, 5 motorcycle mechanics, 3 shop owners, 8 truck pushers, and 5 food sellers) were excluded from analysis because their numbers did not makeup to the required numbers (=10) to be classified as an occupational group as far as this study is a concern. Therefore, 355 total respondents were analyzed: 26 doctors, 37 nurses, 29 MLS, 37 MLT, 21 CHEWs, 15 pharmacists, 47 students, 22 drivers, 19 religious leaders, 48 farmers/villagers, traditional healers 14 and 40 civil servants. The breakdown of the respondents into age and sex is shown in Table 1. The whole study population has a mean age of 36.4 years, within the range of 19-67. Approximately 54% of the HCWs were males, while 63.6% of MCs were males. For HCWs, approximately 30% of them have 1-5 years of working experience while approximately 40 and 20% respectively have 5-10 and 10 and above working experience.

**Table 1:** demographic information of HCWs and MCs recruited for the study

Parameter	HCWs (N=165)	MCs (N=190)
**Age**		
Average (STD)	37.5 (8.0)	35.7 (9.34)
Range	24-57	18-62
**Sex**		
Male (%)	89 (53.9)	121 (63.6)
Female (%)	76 (46.1)	41 (21.5)
Unspecified (%)	0	28 (14.7)
**Year of working experience (years)**		
1-5 (%)	47 (28.5)	33 (17.3)
5-10 (%)	69 (41.8)	51 (26.8)
10-above (%)	32 (19.4)	42 (22.1)
Unspecified/not applicable (%)	17 (10.3)	64 (33.6)

**Knowledge of EVD and AMR:** the participants were asked in the questionnaire about their knowledge of EVD and AMR. Their responses are shown in Table 2. Most HCWs (95.1%) and MCs (82.6%) have heard of EVD before the study. Most of MC´s (92.6%) knowledge is recent (1-5 years) when compared with that of HCWs. The main source of EVD´s information for both HCWs and MCs is media. However, 31.5% of the HCW's source their EVD information in school, but only 9.4% of MCs link their source of EVD information to the school. Further, 16.8% of MCs heard of EVD in their places of worship. Both HCWs and MCs gave reasons like EVD´s way of killing, stigmatization, no drugs, and vaccines as the basis for their knowledge. Alternatively, a lower percentage of HCWs (68.4%) and an extremely low percentage of MCs (9.2%) know AMR before the study (Table 2). While only 4.7% of MCs heard of AMR between 1 and 5 years and only 2.5% heard of AMR in the media, only 17.2% of MCs were aware of AMR as a problem and considered them dangerous. Similarly, approximately 33% of HCWs shared the same view of AMR not that dangerous with MCs. The difference between knowledge of HCWs and MCs on EVD was not statistically significant (P>0.05), but there is a significant difference in the knowledge of the two groups on AMR (p<0.05).

**Table 2:** comparing the knowledge of HCWs and MCs on EVD and ARM

Statement	Distribution (%)			
	EVD		ARM	
	HCWs	MCs	HCWs	MCs
**1) Have you heard of EVD/ARM before?**				
Yes	118 (95.1)	157 (82.6)	113 (68.4)	18 (9.5)
No	3 (2.4)	33 (17.3)	50 (30.3)	144 (76.1)
Not sure	44 (26.6)	0	2 (1.21)	28 (14.7)
**2) How recent have you heard of EVD/ARM?**				
1-5 years	81 (65.3)	176 (92.6)		9 (4.7)
5-10 years	25 (20.1)	12 (6.3)	74 (44.8)	3 (1.5)
10 and above	18 (14.5)	2 (1.05)	38 (23.0)	2 (2.0)
**3) Where is your source of information?**				
School	52 (31.5)	18 (9.4)	74 (44.8)	16 (8.6)
Media	76 (46.0)	120 (63.1)	15 (9.0)	5 (2.5)
Mosque/church	15 (9.0)	32 (16.8)	0	0
Family/friends	22 (13.3)	20 (10.5)	0	2 (1.0)
**4) Did you consider EVD/ARM as dangerous?**				
Yes	108 (65.4)	136 (71.5)	89 (53.9)	33 (17.3)
No	57 (34.6)	34 (17.8)	56 (33.9)	107 (56.3)
I do not know	0	19 (10.0)	20 (12.1)	52 (27.4)
**5) If your answer to 4 above is yes, why did you consider EVD/ARM as dangerous?**				
Their way of killing	70 (42.4)	93 (48.9)	5 (3.0)	0
Stigmatization attached to EVD/ARM	34 (20.6)	26 (13.6)	0 (00.0)	0
Difficulty in treatment	54 (32.7)	55 (28.9)	93 (56.3)	3 (1.5)
No reason	7 (4.2)	16 (8.4)		

**Comparing the dangers of EVD and AMR:** both HCWs and MCs were asked to the best of their knowledge to compare between EVD and AMR, which one could result in higher mortality, spread faster, more dangerous and receive more publicity. Their general responses are presented in Table 3. Approximately 70% of HCWs and 95% of MCs are of the view that EVD results in higher mortality than AMR. However, 43.6% of the total HCWs agreed that AMR is more dangerous than EVD. Alternatively, only 3.6% of them have the belief that AMR is more dangerous than EVD. All the respondents believed that EVD received more publicity than AMR. Differences between the HCWs and MCs in their responses to each question were statistically significant (p<0.05).

**Table 3:** comparing HCWs and MC’s knowledge on the dangers of EVD and ARPs

Code	Comparison questions	Responses		
		HCWs N=165(%)	MCs N=190(%)	Total N=355(%)
1	(a) EVD results in higher mortality	114 (69.0)	179 (94.2)	293 (82.5)
	(b) ARP results in higher mortality	51 (30.9)	11 (5.7)	62 (17.4)
2	(a) EVD spread faster than ARPs	102 (61.8)	190 (100)	292 (82.2)
	(b) ARPs spread faster than EVD	63 (38.1)	0	63 (17.7)
3	(a) EVD is more dangerous than ARPs	93 (56.3)	183 (96.3)	276 (77.7)
	(b) ARP is more dangerous than EVD	72 (43.6)	7 (3.6)	79 (22.2)
4	(a) EVD is more publicized in the media	165 (100)	190 (100)	355 (100)
	(b) ARPs are more publicized in the media	0	0	0 (0.0)

**Transmission of EVD and ARPs:** both HCWs and MCs shared a similar view on means of transmitting EVD (Table 4, Table 5). The HCWs, which include 76.9% doctors, 95.5% nurses, 68.9% MLS, 75.6% MLT, 66.7% pharmacists, and 80.9% CHEW believed that EVD could be transmitted via contact with an EVD infected person. They also believed that EVD could be transmitted through the bodies of an infected dead person, relatives of infected patients, and through the hands of HCWs. Similarly, most HCWs have belief that HCWs and EVD-suspected patients are at high risk of transmitting EVD. Even though only 20.6% of MLS and 32.4% of MLT believed that travelers are at higher risk of EVD, approximately 70% of doctors and 50% of nurses believed that travelers are at high risk of transmitting EVD. All the HCWs shared the same view on places where EVD can be easily contacted. As for ARP transmission, only 60% and 63% of the HCWs believed that ARP can be transmitted via the hand of HCWs and during direct contact with patients, respectively. However, while 8.4% of HCWs have no idea of ARP's method of transmission, 11.5% and 20% of them have no idea of who is at a high risk of transmitting ARPs and where easily ARPs can be contacted, respectively. On the MC's side, the majority shared the same view with HCWs on EVD´s method of transmission and those at high risk of transmitting EVD, but they differ in their beliefs on how ARPs are transmitted. Most MCs (73.6%, 66.3%, and 63.1%) have no idea of ARP's mode of transmission, those at high risk of transmitting ARPs, and where ARPs can be easily contacted, respectively. While only 16.3% attributed ARPs´ spread to contact, approximately 20% believed that ARPs can only be contacted in the hospitals and not in the community.

**Table 4:** comparing different HCW’s knowledge on the transmission of EVD and ARM/ARPs

Question	HCWs						
	Doctors (N=26)	Nurses (N=37)	MLS (N=29)	MLT (N=37)	Pharmacists (N=15)	CHEW (N=21)	Total (N=165)
**1). How does EVD get transmitted?**							
(a) through contact with infected persons	20 (76.9)	35 (95.5)	20 (68.9)	28 (75.6)	10 (66.7)	17 (80.9)	130(78.7)
(b) through bodies of dead person	21 (80.7)	34 (91.8)	20 (68.9)	31 (83.7)	15 (100)	13 (61.9)	134 (81.2)
(c) through touching relatives of infected person	11 (42.3)	30 (81.0)	14 (48.2)	21 (56.7)	12 (80.0)	16 (76.1)	104 (63.0)
(d) through hands of health-care workers	26 (100)	33 (89.1)	21(72.4)	33 (89.1)	15 (100)	20 (95.2)	148 (89.6)
(e) I have no idea	0 (00.0)	0 (00.0)	0 (00.0)	0 (00.0)	0 (00.0)	1 (4.7)	1 (0.6)
**2). Who is at high risk of transmitting EVD?**							
(a) Health-care workers	21 (80.7)	35 (95.5)	23 (79.3)	31 (83.7)	15 (100)	20 (95.2)	145 (87.8)
(b) EVD-suspected patients	26 (100)	37 (100)	26 (89.6)	33 (89.1)	15 (100)	18(85.7)	155 (93.9)
(c) EVD relatives	9 (34.6)	12 (32.4)	4 (13.7)	17 (45.9)	1 (6.6)	15 (71.4)	58 (35.1)
(d) travelers	18 (69.2)	18 (48.6)	6 (20.6)	12 (32.4)	7 (46.6)	9 (42.8)	70 (42.4)
(e) I have no idea	0 (00.0)	0 (00.0)	0 (00.0)	2 (5.4)	0 (00.0)	1 (4.7)	3 (1.8)
**3) Where can EVD be contacted easily?**							
(a) In hospitals	18 (69.2)	33 (89.1)	26 (89.6)	32 (86.4)	12 (80.0)	20 (95.2)	141 (85.4)
(b) In the communities	25 (96.1)	29 (78.3)	22 (75.8)	21 (56.7)	10 (66.7)	19 (90.4)	126 (76.3)
(c) in the market	12 (46.1)	11 (29.7)	15 (51.7)	17 (45.9)	0 (00.0)	3 (14.2)	58 (35.1)
(d) in religious houses	18 (69.2)	23 (62.1)	21 (72.4)	23 (62.1)	2 (13.3)	12 (57.1)	99 (60.0)
(e) funeral gathering	21 (80.7)	19 (51.3)	22 (75.8)	20 (54.0)	11 (73.3)	9 (42.8)	102 (61.8)
**4). How do ARPs get transmitted?**							
(a) through direct contact with patient	21 (80.7)	21 (56.7)	16 (55.1)	18 (48.6)	12 (80.0)	16 (76.1)	104 (63.0)
(b) through bodies of dead person	15 (57.6)	25 (67.5)	2 (6.8)	0 (00.00	3 (20.0)	7 (33.3)	52 (31.5)
(c) through touching relatives of infected person	3 (11.5)	0 (00.0)	7(24.1)	4 (10.8)	3 (20.0)	12 (57.1)	29 (17.5)
(d) through hands of health-care workers	24 (92.3)	17 (45.9)	17 (58.6)	12 (32.4)	11 (73.3)	18 (85.7)	99 (60.0)
(e) I have no idea	0 (00.0	2 (5.4)	5 (17.2)	1 (2.7)	2 (13.3)	4 (19.0)	14 (8.4)
**5). Who is at high risk of transmitting ARPs?**							
(a) Health-care workers	17 (65.3)	28 (75.6)	22 (75.8)	32 (86.4)	15 (100)	17 (80.9)	131 (79.3)
(b) hospitalized patients	21 (80.7)	26 (70.2)	17 (58.6)	24 (64.8)	14 (93.3)	15 (71.4)	117 (70.9)
(c) Relatives of hospitalized patients	13 (50.0)	9 (24.3)	11(37.9)	11 (29.7)	1 (6.6)	3 (14.2)	48 (29.0)
(d) members of community	23 (88.4)	9 (24.3)	19 (65.5)	21 (56.7)	11 (73.3)	12 (57.1)	95 (57.5)
(e) I have no idea	0 (00.0)	3 (8.1)	2 (6.8)	1 (2.7)	7 (46.7)	6 (28.5)	19 (11.5)
**6) Where can ARP be contacted easily?**							
(a) In hospitals	26 (100)	24 (64.8)	19 (65.5)	31 (83.7)	15 (100)	4 (19.0)	119 (72.1)
(b) In the communities	18 (69.2)	16 (43.2)	12 (41.3)	23 (62.1)	12 (80.0)	11 (52.3)	92 (55.7)
(c) in religious houses	10 (38.4)	4 (10.8)	7 (24.1)	11 (29.7)	2 (13.3)	1 (4.7)	35 (21.2)
(d) funeral gathering	3 (11.5)	17 (45.9)	23 (79.3)	10 (27.0)	4 (26.6)	12 (57.1)	69 (41.8)
(e) I have no idea	0	5 (13.5)	9 (31.0)	11 (29.7)	0 (00.0)	8 (38.0)	33 (20.0)

**Table 5:** comparing different MC’s knowledge on the transmission of EVD and ARM

Questions	MCs						
	Students (N=47)	Drivers (N=22)	Religious Leaders (N=19)	Farmers (N=48)	Traditional Healers (N=14)	Civil servants (N=40)	Total (N=190)
**1). How does EVD get transmitted?**							
(a) through contact with infected persons	40(85.1)	22 (100)	15 (78.9)	44 (91.6)	13 (92.8)	39 (97.5)	173 (91.0)
(b) through bodies of dead person	32(68.0)	18 (81.8)	16 (84.2)	46 (95.8)	11 (78.5)	36 (90.0)	159 (83.6)
(c) through touching relatives of infected person	14(29.7)	10 (45.4)	6 (31.5)	32 (66.6)	6 (42.8)	21 (52.5)	89 (46.8)
(d) through hands of health-care workers	31(65.9)	20 (90.9)	16 (84.2)	12 (25.0)	6 (42.8)	32(80.0)	117 (61.5)
(e) I have no idea	16(34.0)	8 (36.3)	6 (31.5)	8 (16.6)	3 (21.4)	2 (5.0)	43 (22.6)
**2). Who is at high risk of transmitting EVD?**							
(a) Health-care workers	21(44.6)	21 (95.4)	17 (89.4)	45 (93.7)	9 (64.3)	29 (72.5)	142 (74.7)
(b) EVD-suspected patients	38(80.8)	20 (90.9)	19 (100)	45 (93.7)	10 (71.4)	33 (82.5)	165 (86.8)
(c) EVD relatives	13(27.6)	13 (59.0)	3 (15.7)	26 (54.1)	0 (00.0)	13 (32.5)	68 (35.7)
(d) Travelers	32(68.0)	18 (81.8)	5 (26.3)	11 (22.9)	1 (7.1)	19 (47.5)	86 (45.2)
(e) I have no idea	9(19.1)	6 (27.2)	6 (31.5)	22 (48.8)	4 (28.5)	27 (67.5)	74 (38.9)
**3) Where can EVD be contacted easily?**							
(a) In hospitals	23(48.9)	21 (95.5)	16 (84.2)	17 (35.4)	10 (71.4)	35 (87.5)	122 (64.3)
(b) In the communities	19(40.4)	14 (63.6)	1 (5.2)	21 (43.7)	9 (64.3)	29 (72.5)	93 (48.9)
(c) in the market	14(29.7)	6 (27.2)	2 (10.5)	7 (14.5)	3 (21.4)	13 (32.5)	45 (23.6)
(d) in religious houses	11(23.4)	17 (77.2)	5 (26.3)	9 (18.7)	0 (00.0)	23 (57.5)	65 (34.2)
(e) funeral gathering	26(55.3)	13 (59.0)	8 (42.1)	2 (4.1)	0 (00.0)	21 (52.5)	70 (36.8)
**4). How do ARPs get transmitted?**							
(a) through direct contact with patient	11 (23.4)	2 (9.0)	4 (21.0)	0 (00.0)	3 (21.4)	11 (27.5)	31 (16.3)
(b) through bodies of dead person	3 (6.3)	4 (18.1)	2 (10.5)	0 (00.0)	0 (00.0)	6 (15.0)	15 (7.8)
(c) through touching relatives of infected person	6 (12.7)	0 (00.0)	4 (21.0)	2 (4.1)	2 (14.2)	5 (12.5)	19 (10.0)
(d) through hands of health-care workers	11 (23.4)	2 (9.0)	7 (36.8)	4(8.3)	2 (14.2)	8 (20.0)	34 (17.8)
(e) I have no idea	34 (72.3)	22 (100)	13 (68.4)	42 (87.5)	5 (45.4)	24 (60.0)	140 (73.6)
**5). Who is at high risk of transmitting ARPs?**							
(a) Health-care workers	14 (29.7)	2 (9.0)	9 (47.3)	6 (12.5)	3 (27.2)	10 (25.0)	44 (23.1)
(b) hospitalized patients	22 (46.8)	0 (00.0)	8 (42.1)	9 (18.7)	0 (00.0)	4 (10.0)	43 (22.6)
(c) Relatives of hospitalized patients	8 (17.0)	0 (00.0)	2 (10.5)	4 (8.3)	0 (00.0)	2 (5.0)	16 (8.4)
(d) members of community	5 (10.6)	1 (4.5)	4 (21.0)	11 (22.9)	1 (9.09)	8 (20.0)	30 (15.7)
(e) I have no idea	29 (61.7)	20 (90.0)	4 (21.0)	37 (77.0)	6 (35.7)	30 (75.0)	126 (66.3)
**6) Where can ARP be contacted easily?**							
(a) In hospitals	9 (19.1)	9 (40.9)	5 (26.3)	6 (12.5)	5 (35.7)	8 (20.0)	42 (22.1)
(b) In the communities	11 (23.4)	4 (18.1)	4 (21.0)	7 (14.5)	1 (7.1)	11 (27.5)	38 (20.0)
(c) in religious houses	5 (10.6)	0 (00.0)	0 (00.0)	10 (20.8)	3 (27.2)	1 (2.5)	18 (9.4)
(d) funeral gathering	7 (14.8)	0 (00.0)	2 (10.5)	7 (14.5)	0 (00.0)	3 (7.5)	19 (10.0)
(e) I have no idea	31 (65.9)	18 (81.8)	16 (84.2)	33 (68.7)	6 (42.8)	16 (40.0)	120 (63.1)

**Prevention and control of EVD and AMR:** responses of the respondents toward the prevention and control of EVD and AMR are shown in Table 6 and Table 7. The differences in responses between HCWs and MCs for questions concerning whether prevention or treatment options should be used to tackle EVD were not significant as all of them agreed that prevention, not treatment is the best option. Similarly, most HCWs (98.1%) and MCs (81.0%) agreed that EVD is better treated with orthodox drugs for now than with traditional medicine. Further, both HCWs and MCs displayed better attitudes toward hand washing and avoidance of contact with infected patients. While approximately 98% of HCWs and 67% of MCs agreed that burial practices should be done with caution, only 51.6% of MCs agreed that avoiding eating bush meat will help to halt the spread of EVD. For AMR prevention, approximately 84% of HCWs and only 17.3% of MCs agreed that careful use of antibiotics can reduce cases of AMR. Of high concern, 84.2% of MCs disagree with the fact that ARP´s treatment is as difficult as is treating EVD and 59.5% of them agreed that traditional medicine could be useful in managing AMR. Unlike HCWs whose majority (73.3%) agreed that regular hand washing could reduce the spread of AMR, many MCs (49.5%) disagree with that. Also, some MCs (59.5%) and HCWs (40.6%) have either agreed or strongly agreed that the use of traditional medicine could be used in treating ARPs.

**Table 6:** comparing the HCW’s and MC’s knowledge on the prevention and control of EVD

Questions	HCWs (N=165)			MCs (N=190)		
	Agree/ Strongly agree	Neutral	Disagree/Strongly disagree	Agree/ Strongly agree	Neutral	Disagree/ strongly disagree
Prevention of EVD is the best way to stop EVD	162 (98.2)	3 (1.8)	0	167 (87.9)	18 (9.5)	5 (2.6)
Treatment of EVD infected patients can prevent spread of EVD	165 (100)	0	0	172 (90.6)	8 (4.2)	10 (5.2)
Orthodox drugs are better than traditional drugs in treating EVD	162 (98.1)	3 (1.8)	0	154 (81.0)	17 (9.0)	19 (10.0)
Traditional medicine can help treat EVD infected patients	61 (36.9)	81 (49.0)	23 (13.0)	122 (64.2)	45 (23.7)	23 (12.1)
Regular hand washing can prevent someone from contacting EVD	159 (96.3)	4 (2.4)	2 (1.2)	101 (53.1)	56 (29.5)	33 (17.4)
Avoiding contact with EVD infected patients can prevent contacting EVD	164 (99.4)	1 (0.6)	0 (0.0)	176 (92.6)	6 (3.2)	8 (4.2)
Avoiding contact with dead bodies during burial can control EVD spread	162 (98.2)	3 (1.8)	0 (0.0)	128 (67.4)	27 (14.2)	35 (18.4)
Avoiding eating bush meats can control EVD spread	131 (79.4)	5 (3.0)	29 (17.6)	98 (51.6)	67 (35.3)	25 (13.1)

**Table 7:** comparing the HCW’s and MC’s knowledge on the prevention and control of ARM

Questions	HCWs (N=165)			MC (N=190)		
	Agree/ Strongly agree	Neutral	Disagree/ strongly disagree	Agree/ Strongly agree	Neutral	Disagree/ strongly disagree
Prudent use of antibiotics is the best way to control the spread of ARP	139 (84.2)	7 (4.2)	19 (11.5)	33 (17.3)	45 (23.7)	112 (59.0)
Treatment of ARP infected patients is as difficult as treating EVD	65 (39.4)	51 (30.9)	49 (29.7)	19 (10.0)	11 (5.8)	160 (84.2)
Traditional drugs can be used in treating ARP	67 (40.6)	34 (20.6)	64 (38.8)	113 (59.5)	23 (12.1)	54 (28.4)
Regular hand washing can prevent someone from contacting ARPs	121 (73.3)	31 (18.8)	13 (7.9)	54 (28.4)	42 (22.1)	94 (49.5)
Avoiding contact with patients hospitalized can prevent contacting ARPs	99 (60.0)	41 (24.8)	25 (15.2)	43 (22.6)	45 (23.7)	102 (53.7)

## Discussion

In this study, a comparison was made among and between different HCWs and MCs regarding their knowledge and beliefs about EVD and AMR/ARPs. This is necessary since before the duo of EVD and ARPs can be curtailed in the community and healthcare settings, a better understanding of HCWs and MC´s knowledge toward EVD (and it´s like such as COVID-19) and AMR is needed. Historically, AMR have occurred for decades; not only in the study area but in the entire country, while the first-ever case of EVD in Nigeria was recorded only in 2014. To the best of our knowledge, this is the first study in the sub-region that compares the awareness level of HCWs and MCs on EVD and AMR. Our study confirms significant gaps in knowledge of AMR among some predominantly educated HCWs and many less educated MCs. The study also revealed that HCW's and MCs´ knowledge on EVD is higher than AMR despite repeated calls by WHO, CDC, and other organizations on the dangers posed by the continuing spread of AMR in hospitals and communities [3,8,12].

Most HCWs and MCs have heard of EVD before the study. This may be connected with the EVD outbreak in Nigeria and neighboring West African countries in 2014. Similar studies not only in Nigeria but in Sierra Leone, Guinea, and Liberia have shown an increased awareness of EVD compared to the pre-2014 EVD outbreak [4,22-24]. It is, however, expected that all HCW's respondents will have a better understanding of EVD and AMR due to their work and the kind of training they passed through compared with MCs whose source of information is only through media. This expectation was confirmed through their responses, where some HCWs have heard of EVD in the last 5-10 years, probably during their professional studies and have precisely based their current knowledge on EVDs´ unique way of killing, the stigma attached to EVD or their families and the fact that no specific drug for its treatment for now. In contrast, only 9.5% of MCs have heard of EVD in the last 1-5 years. Irrespective of the area of specialization or occupation generally, the main source of EVD information for both HCW and MCs is media, which comprises both print and social media. This is in consonance with other reports where both print and social media played a vital role in disseminating information on EVD [25-28]. Approximately 30% of HCWs related their source of EVD information to the school, while religious houses such as mosques and churches are the source of information for about 17% of MCs. Sourcing EVD information by some MCs from mosques and churches may be connected to the appeal made by the Nigerian government to religious organizations to step down congregational activities during outbreaks of major infectious diseases such as EVD and COVID-19. Similar to this, mosques and churches in Sierra Leone had actively participated in building awareness to their members on the disease, its prevention, and assistance for EVD victims [29]. This effort of religious houses critically contributed to cutting in the chain of transmission of EVD through the preaching of safe burial practices [30].

The general knowledge of AMR among MCs and some HCWs was low. Most MCs have never heard of AMR in the last 1-5 years. This is a clear indication that the awareness of the menace of AMR in general and ARPs in specific is low in the community. Even though media houses have been recognized by many as a good source of public information on HIV/AIDs, malaria, cancer, and other related diseases [31], it seems they are not doing enough in educating the public on the dangers of AMR in hospitals and communities. In addition to media houses, none of the MCs has heard of AMR in their religious houses, and approximately 30% and 40% of HCWs and MCs (mainly civil servants and students) respectively heard of AMR in schools. Surprisingly, only a few MCs considered AMR as dangerous. Nevertheless, some HCWs also have a similar belief. Difficulty in treating ARP's infected patients was the main reason given by some 56.9% HCWs, while most MCs have no idea on the AMR mode of killing. Based on the area of specialization, doctors, nurses, MLS, and pharmacists have a higher knowledge of AMR than others such as MLT and CHEW. Previous studies have, however, related areas of specialization with knowledge of certain diseases such as HIV, hepatitis among HCWs [4,32]. However, the type of occupation did not play a significant role in MC's knowledge of AMR. In comparing EVD and AMR, the study showed that a relatively accurate comparison of EVD and AMR dangers depends slightly on the HCW's area of specialization and largely on MC's occupation. EVD was believed to be dangerous by most HCWs (56.3%) and MCs (96.3%), but relatively HCWs and few MCs have contrary beliefs. Those with the contrary may be among those that have believed that the EVD outbreak was a plot by the western countries to reduce the African population. This perception, especially by some MCs, was also reported in other West African countries and has led to attacks on foreign medical and humanitarian workers in the recent past [33]. There is no significant difference in the general belief of HCWs toward EVD dangers, as both HCWs that have direct contact with patients or their products such as doctors, nurses, MLS and those that are not such as pharmacists believed that EVD is dangerous and spread faster. However, significant differences exist between and among the two groups on the dangers of AMR. For both HCWs and MCs to choose EVD as being more dangerous than AMR, while in reality, its dangers are a drop in the ocean compared with AMR, further shows inadequate AMR knowledge, and may account for the major reason why the rates of AMR in our community are getting higher. Available data in the literature have suggested that AMR are more dangerous and have resulted in higher casualties than EVDs [10] [17,18]. With a lack of knowledge and lack of effective surveillance systems and infection control in place, AMR will continue to spread from HCWs to another or patient under their custody.

Both HCWs and MCs strongly believed that effective preventive measures are key to EVD management and not treatment. Few traditional healers still hold to their ancient belief that traditional medicine could be useful. Similar beliefs have been reported elsewhere in some West African countries [34]. This further justified the report of WHO, which says that approximately 70%-80% of the West African population rely on traditional medicine. Strong belief in traditional healers and religious leaders who sometimes falsely claimed to have a cure for the likes of EVD and other difficult to treat infections have in the past fueled the spread of EVD [35]. Such false information with the help of social media and the rumor spreaders can easily reach the ear of several MCs. This study further identified that most HCWs and MCs have moderate to a strong belief that modification of lifestyles by avoiding unsafe burial practices and eating bush meat during epidemics can prevent the spread or control of EVD transmission. This conforms to studies of [4] where willingness to change old habits by HCWs was identified as a predictor of good knowledge. On the AMR, there is a disparity in HCW's and MC's response on prevention and control of AMR spread. More than half of HCWs have the belief that prudent use of antibiotics and hand washing are the best way to control the spread of AMR. Surprisingly, approximately 30% of HCWs disagree with the fact that treatment of ARP infected patients is as difficult as treating EVD. However, the level of knowledge might have affected this option, as most CHEW, MLT, and few MLS and nurses responded in this way (data not shown). Similarly, most HCWs are aware that the avoidance of unprotected contact with a hospitalized patient can prevent contacting ARPs. On the other hand, MCs have contrary beliefs to HCWs. Only 17.3% and 28.4% of MCs believed that caution use of antibiotics and regular hand washing, respectively, is key to combating the spread of AMR. This is a clear indication that there is no adequate knowledge of MCs on the proper use of antibiotics and key infection control techniques.

## Conclusion

Knowledge and awareness of EVD is high among HCWs and MCs, but there is a significant gap in knowledge of AMR in some HCWs and majority of MCs. The study also showed that the majority of HCWs and MCs believe that EVD is more dangerous than AMR and ARPs. While most HCWs believe that prudent use of antibiotics and hand washing are the best way to control the spread of ARPs, some MCs opt for traditional mode of treatment. Heightened publicity and awareness campaigns by government, HCWs, religious leaders, media houses and other stakeholders significantly assisted in increasing MCs knowledge and awareness of EVD and its control in Nigeria. Extending such publicity and awareness campaigns to antimicrobial stewardship programs in hospitals and in communities especially rural areas and among less-educated MCs is highly recommended since the problem of AMR cannot be handled alone by HCWs without the cooperation of MCs and other stakeholders.

### What is known about this topic

- HCW awareness, knowledge and attitude toward EVD in Nigeria;
- Role of media in disseminating EVD awareness in Nigeria;
- Little data on HCW awareness, knowledge and attitude toward ARPs in Nigeria.

### What this study adds

- A significant gap of knowledge of AMR among some predominantly educated HCWs and most less and non-educated MCs in Nigeria was established;
- The heightened rates of AMR in hospitals and communities in Nigeria are because of lack of proper awareness of HCWs and MCs on its danger and it requires urgent public health interventions;
- Public awareness strategies put in place by the government, religious leaders, and media that led to immediate control of the EVD outbreak is needed to slow/stop the spread of AMR.

## References

[1] Okeke I, Sosa A (2003). Antibiotic resistance in Africa-discerning the enemy and plotting a defence. Afr Health.

[2] Team WER (2016). After Ebola in West Africa: Unpredictable Risks, Preventable Epidemics. N Engl J Med.

[3] Word Health Organization (2014). WHO´s first global report on antibiotic resistance reveals serious, worldwide threat to public health. Word Health Organization.

[4] Iliyasu G, Ogoina D, Out AA, Dayyab FM, Ebenso B, Otokpa D (2015). A multi-Site knowledge attitude and practice survey of Ebola Virus Disease in Nigeria. PLoS One.

[5] Priyendu A, Ahmed Z, Nagappa A, Eshwara V,Varma M (2016). Direct costs and length of stay in Carbapenem resistant versus Carbapenem sensitive *Klebsiella pneumonia* infections in a tertiary care hospital. Int J Infect Dis.

[6] Michael Osterholm T, Kristine Moore A, Nicholas Kelley S, Lisa Brosseau M, Gary Wong, Frederick Murphy A (2015). Transmission of Ebola viruses: what we know and what we do not know. MBio.

[7] Weber D, Rutala W, Kanamori H, Gergen MF,Sickbert-Bennett EE (2015). Carbapenem-resistant Enterobacteriaceae: frequency of hospital room contamination and survival on various inoculated surfaces. Infect Cont Hosp Epid.

[8] Word Health Organization Antibiotic resistance fact sheet. Word Health Organization.

[9] Shittu AO, Okon K, Adesida S, Oyedara O, Witte W, Strommenger B (2011). Antibiotic resistance and molecular epidemiology of *Staphylococcus aureus* in Nigeria. BMC Microbiol.

[10] Yusuf I, Adam RU, Ahmad SA, Yee PL (2014). Ebola and compliance with infection prevention measures in Nigeria. Lancet Infect Dis.

[11] Mckenna M (2014). Antibiotic resistance fact sheet.

[12] Center for Desease Control (2016). 2014-2016 Ebola Outbreak in West Africa. Center for Desease Control.

[13] Gurieva TV, Bootsma MC, Bonten MJ (2012). Decolonization of patients and health care workers to control nosocomial spread of methicillin-resistant *Staphylococcus aureus* a simulation study. BMC Infect Dis.

[14] Olushayo Olu, Brima Kargbo, Sarian Kamara, Alie H Wurie, Jackson Amone, Louisa Ganda (2015). Epidemiology of Ebola virus disease transmission among health care workers in Sierra Leone, May to December 2014: a retrospective descriptive study. BMC Infect Dis.

[15] Plipat N, Spicknall IH, Koopman JS, Eisenberg JN (2013). The dynamics of methicillin-resistant *Staphylococcus aureus* exposure in a hospital model and the potential for environmental intervention. BMC Infect Dis.

[16] Yusuf I, Arzai A, Haruna MI, Sharif AA, Getso MI (2014b). Detection of multi drug-resistant bacteria in major hospitals in Kano, North-West, Nigeria. Braz J Microbiol.

[17] O´Malley SM, Emele FE, Nwaokorie FO, Idika N, Umeizudike AK, Emeka-Nwabunnia I (2015). Molecular typing of antibiotic-resistant *Staphylococcus aureus* in Nigeria. J Infect Public Health.

[18] Oduyebo O, Falayi O, Oshun P, Ettu A (2015). Phenotypic determination of carbapenemase producing *Enterobacteriaceae* isolates from clinical specimens at a tertiary hospital in Lagos, Nigeria. Niger Postgrad Med J.

[19] Gupta N, Mehta N, Gupta P, Arora V, Setia P (2015). Knowledge regarding Ebola Hemorrhagic Fever among private dental practitioners in Tricity, India: a cross-sectional questionnaire study. Niger Med J.

[20] Unnati Patel, Jennifer Pharr, Chidi Ihesiaba, Frances Oduenyi, Aaron Hunt, Dina Patel (2016). Ebola outbreak in Nigeria: increasing Ebola knowledge of volunteer health advisors. Glob J Health Sci.

[21] Shittu RO, Sanni MA, Odeigah LO, Akanbi AA, Sule AG, Isiaka-Lawal S (2015). Awareness, knowledge and misconceptions about Ebola Virus Disease (EVD) in a family practice setting in Nigeria, West Africa. J Antivir Antiretrovir.

[22] Jiang H, Shi GQ, Tu WX, Zheng CJ, Lai XH, Li XX (2016). Rapid assessment of knowledge, attitudes, practices, and risk perception related to the prevention and control of Ebola virus disease in three communities of Sierra Leone. Infect Dis Pove.

[23] Kobayashi M, Beer K, Bjork A, Chatham-Stephens K, Cherry CC, Arzoaquoi S (2015). Community Knowledge, attitudes, and practices regarding Ebola Virus Disease-Five Counties, Liberia, September-October, 2014. MMWR Morb Mortal Wkly Rep.

[24] Paul Pronyk, Braeden Rogers, Sylvia Lee, Aarunima Bhatnagar, Yaron Wolman, Roeland Monasch (2016). The effect of community-based prevention and care on Ebola transmission in Sierra Leone. Ame J Pub Health.

[25] Chapman H, Animasahun V, Tade A, Asad Naveed (2016). Addressing the role of medical students using community mobilization and social media in the Ebola response. Perspect Med Edu.

[26] Li Q, Lu F, Dai C, Fan M, Wang W, Wang K (2017). Simulating the potential role of media coverage and infected bats in the 2014 Ebola outbreak. J Theor Biol.

[27] Sell TK, Boddie C, McGinty EE, Pollack K, Smith KC, Burke TA (2016). News media coverage of US Ebola policies: implications for communication during future infectious disease threats. Prev Med.

[28] Smith S, Smith S (2016). Media coverage of the Ebola virus disease in four widely circulated Nigerian newspapers: lessons from Nigeria. Heal Promot Perspect.

[29] World Council of Churches (2014). World Council of Churches expresses deep solidarity on Ebola. Vatican Radio.

[30] World vision (2014). The Battle to Overcome Ebola in West Africa. Word Vision.

[31] Yusuf I, Adam RU (2014). Adopting Chennai declaration strategies in the prevention and control of the spread of multidrug-resistant hospital-acquired bacterial infections in Nigeria: a call to action. J Glob Antimicrob Resist.

[32] Zafar A, Aslam N, Nasir N, Meraj R, Mehraj V (2008). Knowledge, attitudes and practices of health care workers regarding needle stick injuries at a tertiary care hospital in Pakistan. J Pak Med Assoc.

[33] Karamouzian M, Hategekimana C (2015). Ebola treatment and prevention are not the only battles: understanding Ebola-related fear and stigma. Int J Health Pol Manage.

[34] Eplett (2015). For Ebola, traditional Healers and a more exclusive approach, Scientific American.

[35] Manguvo A, Mafuvadze B (2015). The impact of traditional and religious practices on the spread of Ebola in West Africa: time for a strategic shift. The Pan Afr Med J.

